# High-Precision Control of Aviation Photoelectric-Stabilized Platform Using Extended State Observer-Based Kalman Filter

**DOI:** 10.3390/s23229204

**Published:** 2023-11-15

**Authors:** Lu Wang, Xiantao Li, Yuzhang Liu, Dapeng Mao, Bao Zhang

**Affiliations:** 1 University of Chinese Academy of Sciences, No.19, Yuquan Rd., Beijing 100049, China; wanglu8775@163.com; 2 Changchun Institute of Optics, Fine Mechanics and Physics, Chinese Academy of Sciences, Changchun 130033, China

**Keywords:** extended state observer (ESO), Kalman filter (KF), gyro noise, disturbance rejection

## Abstract

The accuracy of the line-of-sight of aviation photoelectric optoelectronic stabilization platforms is limited by two factors: external disturbance and sensor noise. An extended state observer (ESO) can effectively improve their anti-interference ability. However, due to the serious problem of gyroscope noise, further improvement of an ESO’s disturbance suppression effect is limited. This article proposes a control structure that combines a Kalman filter (KF) and ESO, effectively improving upon the interference suppression ability of a traditional ESO under the influence of noise. Firstly, an ESO was used to observe the lumped disturbance of the system, and then, the observed disturbance was compensated for in the control loop. Secondly, based on the compensation servo control system, the state equation of the system was reconstructed using a Kalman filter. Finally, the reconstructed filtered state variables were iterated onto the universal state observer, achieving the observation of disturbances while filtering out sensor noise. Under the conditions of a laboratory flight simulation turntable, the line-of-sight stability accuracy level was improved under disturbance excitation. It can be seen that the combination of a Kalman filter and extended disturbance observer proposed in this project improves the ESO’s anti-interference ability under the influence of noise.

## 1. Introduction

**Figure 1:**
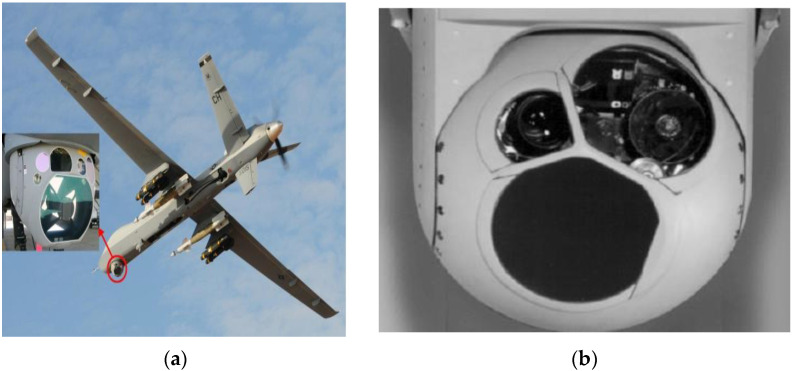
Unmanned aerial vehicles and airborne optoelectronic stability platforms: (**a**) a drone loaded with an optoelectronic device and (**b**) an airborne photoelectric stabilized platform.

An airborne optoelectronic stabilization platform refers to the device used in carrier systems, which uses a gyroscope as the sensitive element and a servo control algorithm to isolate the carrier disturbance, so as to maintain the stability of the detector’s visual axis in inertial space and realize the functions of target acquisition, tracking, and aiming [1,2]. Figure 1 shows the schematic diagram of UAV and airborne optoelectronic platform.

LOS stability accuracy is an important technical indicator of the airborne optoelectronic platform. The attitude change of the carrier will directly affect the LOS pointing of the system through the friction torque and mass imbalance torque when the carrier flies during a large maneuver under a complex electromagnetic environment. At the same time, the complex electromagnetic environment causes the gyroscope and other sensor elements to produce large electromagnetic noise, which together with the disturbance will lead to the LOS jitter of the optoelectronic platform, thus in turn leading to imaging blur, image jitter, and other phenomena of the detector, which may even deviate from the detection field-of-view of the system, leading to tracking failure [3,4,5,6].

Using a disturbance observer (DOB) and active disturbance rejection control (ADRC) has been widely studied and applied. Both can effectively handle the impact of uncertainty in disturbance models on control performance.

The frequency domain design method of the DOB was proposed by Japanese scholar Ohnishi in 1987. Its basic design idea was to equate the differences between the actual object and the nominal model caused by external interference and model parameter changes to the control input, in order to observe the disturbance [7]. A DOB has been successfully applied in many industrial applications, such as servo systems [8,9], robot systems [10], and torque control systems [11]. In order to improve the performance of the traditional DOB and reduce the burden of disturbance observation, scholars have proposed an estimation strategy combining a neural network (NN) with the DOB in recent years. An NN is used to approximate uncertain internal dynamics, and the DOB is used for estimation of time-varying external disturbances [12,13,14]. The inertially stabilized platform is affected by its usage environment, and the mathematical model may fluctuate under different operating conditions. The DOB estimates and compensates for disturbances based on the inverse model of the controlled system, which limits its application on an airborne optoelectronic stabilization platform.

Active disturbance rejection control (ADRC) is a control method proposed by Chinese scholar Jingqing Han in 1995 [15]. This method does not require knowing the exact mathematical model of the controlled object. In response to the problem of complex and diverse sources of disturbance to the controlled object, ADRC treats the internal and external disturbances to the controlled object as lumped disturbances and uses an extended state observer (ESO) to estimate and compensate for the disturbances [16,17,18,19,20,21]. In order to further reduce the difficulty of parameter debugging for nonlinear active disturbance rejection controllers, Professor Gao Zhiqiang from Cleveland University in the United States proposed an improved ADRC method. This method changes the nonlinear combination in the ADRC to a PD controller and uses linear functions to replace the nonlinear functions in the original control structure. The improved linear ADRC has the advantages of good disturbance rejection performance, strong robustness, high accuracy, and synchronous estimation of a system’s state and disturbance. It has high engineering application value in airborne optoelectronic stabilization platforms [22,23,24].

In order to improve the accuracy and real-time performance of traditional ESOs for disturbance observation, it is usually necessary to select the gain of the active disturbance rejection controller to be as high as possible. However, in the actual usage environment of airborne optoelectronic platforms, the noise of sensors always exists. In complex electromagnetic environments, the noise of sensors become more severe. An ESO controller amplifies the noise of the sensor, leading to controller saturation and control performance decline [25], and even causing instability [26,27].

The complex environment of airborne optoelectronic platforms, as well as factors such as vibration and airflow disturbances of aircraft carriers, further exacerbate signal noise, reduce the bandwidth of control systems, and limit the further improvement in line-of-sight stability accuracy [28,29]. There are mainly low-pass filtering methods [30], wavelet filtering methods [31,32], and information fusion processing and Kalman filter methods to resolve this. These methods directly process the angular velocity signal output by the gyroscope. Due to various factors such as aircraft body vibration and airflow disturbances, the noise frequency of airborne optoelectronic platforms is complex and there is no determined spectrum. When low-pass filtering filters out high-frequency noise, the low-frequency useful signal of the sensor is also attenuated. The wavelet filtering algorithm is complex and has poor real-time performance. Airborne optoelectronic platforms require real-time compensation for external interference, making it difficult to apply the wavelet filtering algorithm and even causing system instability; in recent years, scholars such as Luo Yong and Guo Hui have applied information fusion technology to gyroscope data processing, using the design of multiple gyroscopes and accelerometers to fuse the data to obtain virtual gyroscope data that filter out noise and zero drift, for use in velocity loops [33,34,35]. Moreover, filtering inevitably leads to lag, which also limits the bandwidth of the ESO controller [36,37].

In summary, traditional extended state observers (ESOs) cannot handle sensor noise when observing disturbances, resulting in noise coupling into the control loop and reducing the accuracy of disturbance observation. Without considering platform disturbances, noise filtering algorithms based solely on the mathematical model of gyroscopes cannot achieve good results. Based on the above reasons, exploring an algorithm combining disturbance suppression and noise processing, improving the controller bandwidth of the ESO, and further reducing disturbances are of great significance in the practical application of anti-interference control performance.

At present, research on the stability accuracy of airborne optoelectronic platforms involve reducing gyro noise and increasing controller bandwidth; on the other hand, through the study of anti-interference algorithms, the impact of disturbances on the stable circuit is reduced. However, few studies have effectively combined the two methods. Noise and disturbance interact with each other, limiting the bandwidth improvement of anti-interference algorithms. Disturbance also exacerbates gyro noise. Therefore, it is necessary to study the combination of noise processing and disturbance suppression. This article combines the ESO disturbance observation algorithm with the Kalman filter algorithm, using an ESO observer to observe a disturbance and compensate it into the control loop. The Kalman filter algorithm was used to process the velocity signal of the control system after disturbance compensation to eliminate gyro noise. A Kalman filter reduces sensor noise, and an ESO controller can further increase gain, making its disturbance observation more accurate. The ESO eliminates disturbances more accurately, making the Kalman filter control model more accurate, which can further improve the effectiveness of Kalman filter in reducing noise. Combining ESO observation disturbance with a Kalman filter to eliminate noise and iterate in this way can therefore achieve a better disturbance suppression effect.

In order to improve the stability of the line-of-sight of airborne optoelectronic platforms and improve the problem of a limited disturbance suppression effect in traditional ESOs when noise is severe, this paper makes the following contributions:

1. After analysis, it was concluded that disturbance suppression of airborne optoelectronic platforms must be carried out simultaneously with noise processing, and an ESO + KF control method was proposed.
2. An algorithm derivation and the design of airborne optoelectronic platform servo control system were performed.
3. Simulating the flight environment in the laboratory proved that the algorithm has a better disturbance suppression effect.

## 2. Materials and Methods

**Figure 2:**
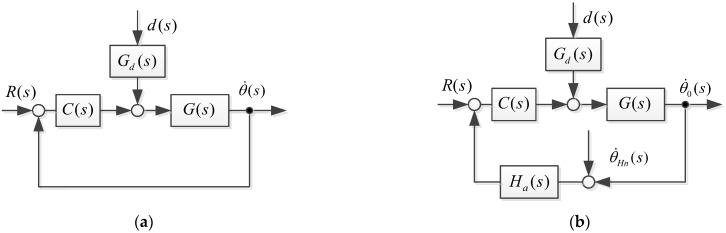
Inertial stability system control diagram (**a**) under ideal conditions and (**b**) considering noise and time delay.

The servo control circuit of the airborne optoelectronic stabilization platform is affected by combined disturbance and gyro noise, which limits the further improvement of its line-of-sight stability accuracy. The model and analysis of the stable platform are shown in Figure 2.

In the control system block diagram of Figure 2a, G(s) is the mathematical model of inertial stability platform, C(s) is the speed loop controller, R(s) is the input of the controller system, d(s) is the disturbance of the system, $G_{d}(s)$ is the transfer function of the disturbance, and $\dot{\theta }\left(s\right)$ is the angular velocity of the gyroscope under ideal system conditions.

Due to the vibration of the carrier and the influence of the external environment, the noise of the gyroscope is severe. The inertial stability system control diagram considering noise and time delays is shown as Figure 2b, where ${\dot{\theta }}_{Hn}(s)$ is the high frequency noise of the gyro, $H_{a}(s)$ is the transfer function of the gyroscopic measurement of angular velocity, and ${\dot{\theta }}_{0}\left(s\right)$ is the angular velocity of the gyroscope in an actual situation.

An ideal situation, without considering the low-pass filtering and delay characteristics of the gyroscope, is shown in Figure 2a. The transfer function of the gyroscope is approximately equivalent to 1 and the ideal system output is

(1) $$\dot{\theta }(s)=\frac{C(s)\cdot G(s)}{1+C(s)\cdot G(s)}\cdot R(s)+\frac{G_{d}(s)\cdot G(s)}{1+C(s)\cdot G(s)}\cdot d(s)$$

Due to the limitations of the operating environment of the airborne optoelectronic platform, high-frequency vibrations of the aircraft can be transmitted to the frame, and airborne electromagnetic noise is relatively severe. Therefore, in addition to platform motion, the gyroscope also contains high-frequency noise. The measured values of the gyroscope can be expressed as the following:

(2) $${\dot{\theta }}_{0}(s)=\frac{C(s)\cdot G(s)}{1+C(s)\cdot G(s)\cdot H_{a}(s)}\cdot R(s)+\frac{G_{d}(s)\cdot G(s)}{1+C(s)\cdot G(s)\cdot H_{a}(s)}\cdot d(s)+\frac{C(s)\cdot G(s)\cdot H_{a}(s)}{1+C(s)\cdot G(s)\cdot H_{a}(s)}\cdot {\dot{\theta }}_{Hn}(s)$$

where

(3) $$H_{a}(s)=\frac{k_{a}\omega_{c}}{s+\omega_{c}}e^{-T_{d}s}$$

and $\omega_{c}$ is the gyro measurement bandwidth and $T_{d}$ is the gyro measurement delay time.

When the inertially stabilized platform is in a stable state and the input is 0, the equation is the following:

(4) $${\dot{\theta }}_{0}(s)=\frac{G_{d}(s)\cdot G(s)}{1+C(s)\cdot G(s)\cdot H_{a}(s)}\cdot d(s)+\frac{C(s)\cdot G(s)\cdot H_{a}(s)}{1+C(s)\cdot G(s)\cdot H_{a}(s)}\cdot {\dot{\theta }}_{Hn}(s)$$

According to the above equation analysis, the impacts of gyroscopes and disturbances on the inertially stabilized platform of the system are the following:

1. When the system input is 0, both external disturbances and high-frequency noise from the gyroscope will affect the stability accuracy of the platform.
2. Reducing the impact disturbances on the output of the control system and improving stability accuracy can be achieved by increasing the gain of the controller C(s). However, the high-frequency noise of sensors can bring high-frequency components to the control loop, affecting stability accuracy. In severe cases, it may even cause platform vibration. As the frequency increases, $1+C(s)\cdot G(s)\cdot H_{a}(s)$ decreases. This exacerbates the impact of high-frequency noise on the system and limits the improvement in controller gain. Therefore, the processing of gyroscope noise cannot be ignored while suppressing disturbance.
3. When the external disturbance frequency of the system is greater than the bandwidth of the gyro, as the frequency increases, *H_a_*(*s*) will decrease, resulting in a decrease in $1+C(s)\cdot G(s)\cdot H_{a}(s)$. This weakens the ability of the inertially stabilized platform to suppress disturbance. When processing the gyro signal, the bandwidth of the gyro cannot be reduced.
4. The delay of the gyroscope will affect the close loop bandwidth and high-frequency gain of the inertially stabilized platform, thereby affecting the disturbance suppression ability and stability accuracy. When processing gyroscope data, there should be no excessive delay.

In summary, the stability accuracy of the inertial stabilization platform is not only affected by external disturbances, but also by gyro noise. Gyro noise amplifies the high-frequency components in the disturbance. The high-frequency noise of the gyroscope brings high-frequency components in the control loop, limiting the improvement of controller bandwidth and reducing disturbance suppression ability. Therefore, in order to further improve the accuracy of line-of-sight stability, disturbance compensation and the reduction of sensor high-frequency noise are necessary.

The airborne optoelectronic platform system is a single-output single-output system, which is suitable for an n-order single-input single-output system [38]:

(5) $$x^{n}=f(x,\dot{x},\cdots ,x^{n-1},w,t)+bu$$

where $x,\overset{\cdot }{x},\cdots ,x^{n-1}$, respectively, represents the state of the system and its differential orders and $f(x,\overset{\cdot }{x},\cdots ,x^{n-1},w,t)$ is the lumped disturbance that combines external and internal disturbance. The key to ESOs lies in the real-time estimation of $f(x,\overset{\cdot }{x},\cdots ,x^{n-1},w,t)$ and eliminating it. But this process does not consider the impact of noise.

The ESO algorithm expands the lumped disturbance into a new state variable of the system:

(6) $$x_{n+1}=f(x_{1},\cdots ,x_{n},w(t),t)$$

Then, the original state variables and disturbance states are observed using the system output, and the extended state equation is the following:

(7) $$\left\{\begin{array}{l} {\dot{x}}_{1}=x_{2}\\ {\dot{x}}_{2}=x_{3}\\ \vdots \\ {\dot{x}}_{n}=x_{n+1}+bu\\ y=x_{1} \end{array}\right.$$

Establish a linear observation equation based on the state equation as the following:

(8) $$\left\{\begin{array}{l} {\dot{z}}_{1}=z_{2}-\beta_{1}(z_{1}-y)\\ {\dot{z}}_{2}=z_{3}-\beta_{2}(z_{1}-y)\\ \vdots \\ {\dot{z}}_{n}=z_{n+1}-\beta_{n}(z_{1}-y)+bu\\ {\dot{z}}_{n+1}=-\beta_{n+1}(z_{1}-y)\\ y=z_{1} \end{array}\right.$$

The error state equation is

(9) $$\left\{\begin{array}{l} {\dot{e}}_{1}=e_{2}-\beta_{1}e_{1}\\ {\dot{e}}_{2}=e_{3}-\beta_{2}e_{1}\\ \vdots \\ {\dot{e}}_{n}=e_{n+1}-\beta_{n}e_{1}\\ {\dot{e}}_{n+1}=-\beta_{n+1}e_{1}-h(t)\\ y=z_{1} \end{array}\right.$$

Transforming the ESO equation into the form of a state equation gives

(10) $$\left\{\begin{array}{l} e_{1}(k)=e_{1}(k-1)-Te_{2}(k-1)-T\beta_{1}e_{1}\\ e_{2}(k)=e_{2}(k-1)-Te_{3}(k-1)-T\beta_{2}e_{1}\\ \vdots \\ e_{n}(k)=e_{n}(k-1)-Te_{n+1}(k-1)-T\beta_{n}e_{1}\\ e_{n+1}(k)=e_{n+1}(k-1)-Te_{n+1}e_{1}-Th(t)\\ y=x_{1} \end{array}\right.$$

**Figure 3:**
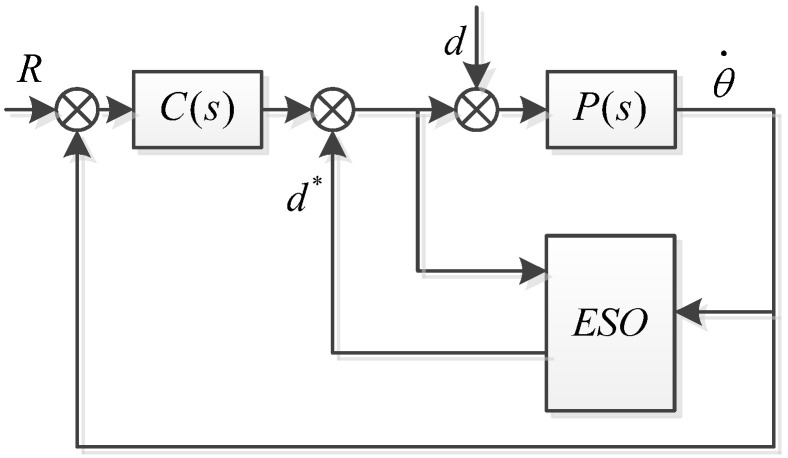
Inertial stability platform system control diagram.

The basic logic of an ESO is shown in Figure 3. The ESO observes external disturbances and compensates them in real-time into the control loop.

The traditional Kalman filter equation is the following:

(11) $$\left\{\begin{array}{l} x_{k}=Ax_{k-1}+Bu_{k}+w_{k}\\ y_{k}=Cx_{k}+v_{k} \end{array}\right.$$

where $x_{k}$ is the state variable, $w_{k}$ is the process noise, $v_{k}$ is measurement noise, and $w_{k}$ and $v_{k}$ are white noise that satisfy the Gaussian distribution.

The Kalman filter uses the optimal result from the previous moment to predict the predicted value at the present moment and uses the observed value at the present moment to correct the predicted value at the present moment to obtain the optimal result. The operation process is as follows [39].

The prediction equation is

(12) $$\left\{\begin{array}{l} \overset{\wedge }{x_{t}^{-}}=F\overset{\wedge }{x_{t-1}^{-}}+Bu_{t-1}\\ P_{t}^{-}=FP_{t-1}F^{T}+Q \end{array}\right.$$

The updated equation is

(13) $$\left\{\begin{array}{l} K_{t}=P_{t}^{-}H^{T}(HP_{t}^{-}H^{T})\\ \overset{\wedge }{x_{t}}=\overset{\wedge }{x_{t}^{-}}+K_{t}(Z_{t}-H\overset{\wedge }{x_{t}^{-}})\\ P_{t}=(I-K_{t}H)P_{t}^{-} \end{array}\right.$$

**Figure 4:**
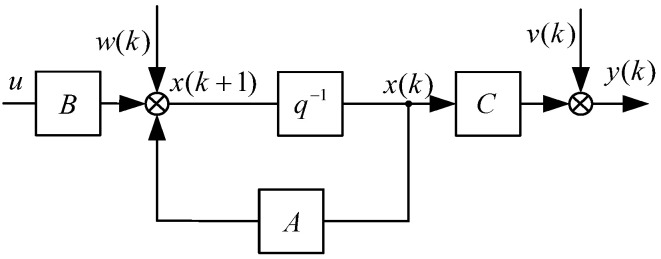
Basic flowchart of the Kalman filter.

The basic process of the Kalman filter is shown in Figure 4.

When designing platform control systems using a traditional Kalman filter, the influence of disturbances is not considered. The main factor that affects the stability accuracy of the airborne optoelectronic platform’s line-of-sight is disturbance, which comes from various sources, including low-frequency friction, wind resistance, and high-frequency body vibration. This needs to be carefully considered. Improvements need to be made on the traditional Kalman filter architecture.

## 3. Proposed ESO + KF

This section is divided by subheadings. It should provide a concise and precise description of the experimental results, their interpretation, as well as the experimental conclusions that can be drawn.

In order to achieve long focal distance tracking of airborne optoelectronic platforms, the accuracy of line-of-sight stability is one of the most important indicators. The noise of the gyroscope and the disturbance of the airborne usage environment are the two main factors that affect the accuracy of line-of-sight stability. Traditional ESOs only compensate for disturbances and do not consider the impact of gyro noise. In the usage environment of air-borne optoelectronic stabilization platforms, gyro noise is severe, and not handling gyro noise will result in a poor disturbance suppression effect.

This article proposes an ESO + KF algorithm. This algorithm uses a Kalman filter to process gyro noise on the basis of traditional ESO compensation disturbance. This ESO + KF algorithm is less affected by noise, has more accurate disturbance compensation, and has smaller speed fluctuations. The specific principle of this algorithm is as follows.

A single-input single-output system affected by external disturbances and noise can be represented as the following:

(14) $$\left\{\begin{array}{l} x_{k}=Ax_{k-1}+Bu_{k-1}+D\cdot d_{k-1}+w_{k-1}\\ y_{k}=Cx_{k}+v_{k} \end{array}\right.$$

where $d_{k-1}$ is the disturbance of the inertially stabilized platform, which is a type of disturbance that belongs to low-frequency disturbance. $w_{k-1}$ is the process noise of systems that cannot contain lumped disturbances, which is a type of disturbance that belongs to high-frequency noise. $x\in R^{n},u\in R,y\in R^{r},v\in R^{n}$ are the state variables, system inputs, system outputs, and measurement noise, respectively.

The above equation $d_{k-1}$ can be observed by an extended state observer, which can counteract the observed disturbance values with external disturbances. When the disturbance observation effect is accurate, the error is approximately 0, which can eliminate the influence of disturbances on the stable circuit. However, due to various limitations, disturbance observations inevitably have errors, and the errors of disturbance observations are bounded. The residual of disturbance observations can be further eliminated through a Kalman filter.

For the above system, the following assumptions were made.

**Assumption** **1.** w *and* v *are white noise that satisfies the Gaussian distribution.*

**Assumption** **2.** *The lumped perturbation* d *is bounded and the derivative of* d *is bounded.*

**Assumption** **3.** *Fast changing disturbance* w *and measurement noise* v *are bounded.*

**Assumption** **4.** *When in steady state,* d*, is a constant value, the derivative of it is 0, and the second derivative of* d *is 0.*

(15) $$\left\{\begin{array}{l} x_{k}=Ax_{k-1}+Bu_{k-1}+D\cdot (d_{k-1}-{\hat{d}}_{k-1})+w_{k-1}\\ y_{k}=Cx_{k}+v_{k} \end{array}\right.$$

(16) $$\left\{\begin{array}{l} x_{k}=Ax_{k-1}+Bu_{k-1}+D\cdot {\tilde{d}}_{k-1}+w_{k-1}\\ y_{k}=Cx_{k}+v_{k} \end{array}\right.$$

*where* $w_{k-1}$ *is the disturbance residual.*

If p(w)∼(0,S), p(v)∼(0,R), p(v)~(0, R), then write the Kalman filter algorithm based on the state equation as the following:

(17) $$\left\{\begin{array}{l} \overset{\wedge }{x_{k}^{-}}=A\cdot \overset{\wedge }{x_{k-1}^{-}}+B\cdot u_{k-1}\\ {\overset{\wedge }{x}}_{kmea}=y_{k}\cdot C^{-1} \end{array}\right.$$

The formula for obtaining the Kalman filter is

(18) $${\hat{x}}_{k}={\hat{x}}_{k}^{-}+K\cdot (y_{k}-C\cdot {\hat{x}}_{k}^{-})$$

where ${\hat{x}}_{k}$ is the observed output by the Kalman filter in step k. When K=0, this indicates that the estimation result is accurate and the measurement error is large, and the output result is completely determined by the estimated prior value. When K=1, this indicates that the estimated value is inaccurate, while the measurement noise is small, and the credibility of the measurement results is high. The output value is completely determined by the measurement results. Continuously adjust the value of K and update weights between estimated and measured values.

Derive the ESO + KF algorithm.

(19) $$P_{k}=E\left[e\cdot e^{T}\right]$$

where $e=x_{k}-{\hat{x}}_{k}$ is the observation error, then substitute Equation (3) into Equation (4).

(20) $$e=(I-K\cdot C)\cdot (x_{k}-{\hat{x}}_{k}^{-})-K\cdot V_{k}$$

Substitute Equation (21) into Equation (20).

(21) $$P_{k}=E\left[e\cdot e^{T}\right]=P_{k}^{-}-P_{k}^{-}C^{T}K^{T}-KCP_{k}^{-}+KCP_{k}^{-}C^{T}K^{T}+KRK^{T}$$

(22) $$tr(P_{k})=tr(P_{k}^{-})-2tr(KCP_{k}^{-})+tr(KCP_{k}^{-}C^{T}K^{T})+tr(KRK^{T})$$

(23) $$\frac{tr(P_{k})}{dK}=-2{\left(CP_{k}^{-}\right)}^{T}+2KCP_{k}^{-}C^{T}+2KR=0$$

Derived from Equations (22) to (24), the Kalman gain is the following:

(24) $$K=\frac{P_{k}^{-}\cdot C^{T}}{C\cdot P_{k}^{-}\cdot C^{T}+R}$$

(25) $$P_{k}^{-}=E\left[e_{k}^{-}\cdot e_{K}^{-T}\right]$$

(26) $$e_{k}^{-}=x_{k}-{\hat{x}}_{k}^{-}=Ax_{k-1}+Bu_{k-1}+Dd_{k-1}+w_{k}-(A{\hat{x}}_{k-1}^{-}+Bu_{k-1}+D{\hat{d}}_{k-1})$$

(27) $$\begin{array}{l} P_{k}^{-}=E(Ae_{k-1}e_{k-1}^{T}A^{T})+E(Ae_{k-1}{\tilde{d}}_{k-1}^{T}D^{T})+E(Ae_{k-1}w_{k-1}^{T})+E(D{\tilde{d}}_{k-1}^{T}e_{k-1}^{T}A^{T})\\ +E(D{\tilde{d}}_{k-1}{\tilde{d}}_{k-1}^{T}D^{T})+E(D{\tilde{d}}_{k-1}w_{k-1}^{T})+E(w_{k-1}e_{k-1}^{T}A^{T})+E(w_{k-1}{\tilde{d}}_{k-1}^{T}D^{T})+E(w_{k-1}w_{k-1}^{T}) \end{array}$$

(28) $$\tilde{d}=d-\hat{d}$$

(29) $$p(\tilde{d})∼(0,Q)$$

And $e_{k}=x_{k}-{\hat{x}}_{k}=Ax_{k-1}+Bu_{k-1}+Dd_{k-1}+w_{k-1}-\left[{\hat{x}}_{k}^{-}+K\cdot (y_{k}-C\cdot {\hat{x}}_{k}^{-})\right]$, so $e_{k}$ and $w_{k}$ are not related, the mathematic expectations of $e_{k}$ and $w_{k}$ are 0, and $E(Ae_{k-1}w_{k-1}^{T})=0$ and $E(w_{k-1}e_{k-1}^{T}A^{T})=0$ can be derived.

${\tilde{d}}_{k}=d_{k}-{\hat{d}}_{k}=d_{k}-\left[{\hat{d}}_{k-1}-fal(e_{k-1})\right]$, where ${\tilde{d}}_{k}$ is uncorrelated with $w_{k}$, $E({\tilde{d}}_{k})=0$, $E(w_{k})=0$, and $E(w_{k-1}{\tilde{d}}_{k-1}^{T}D^{T})=0$, $E(D{\tilde{d}}_{k-1}^{T}w_{k-1}^{T})=0$.

${\tilde{d}}_{k}$ is disturbance observation error, $e_{k}$ is state observation error, and ${\tilde{d}}_{k}$ is uncorrelated with $e_{k}$, which leads to

(30) $$P_{k}^{-}=AP_{k-1}A^{T}+S+DQD^{T}$$

The five formulas for deriving ESO + KF are

(31) $$\left\{\begin{array}{l} {\hat{x}}_{k}^{-}=A\cdot {\hat{x}}_{k-1}+B\cdot u_{k-1}+D\cdot {\hat{d}}_{k-1}\\ P_{k}^{-}=AP_{k-1}A^{T}+S+DQD^{T} \end{array}\right.$$

(32) $$\left\{\begin{array}{l} K=\frac{P_{k}^{-}\cdot C^{T}}{C\cdot P_{k}^{-}C^{T}+R}\\ {\hat{x}}_{k}={\hat{x}}_{k}^{-}+K(y_{k}-C{\hat{x}}_{k}^{-})\\ P_{k}=P_{k}^{-}(I-K\cdot H) \end{array}\right.$$

**Figure 5:**
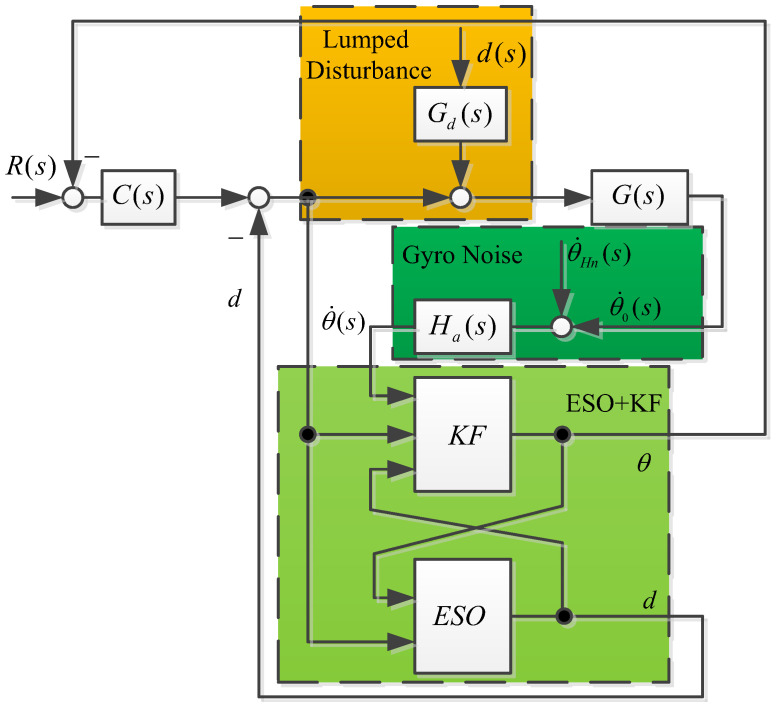
ESO + KF algorithm control block diagram.

The logic diagram of the control system using an ESO combined with a Kalman filter is shown in Figure 5.

By using the above five formulas, signal noise can be filtered out, and the filtered signal can be perturbed by ESO observations. At the same time, the bandwidth of the ESO is higher, and the observed disturbances are more accurate. This further improves the ability of Kalman filter observation speed, making the final ESO + KF algorithm’s observation speed and disturbances more accurate.

## 4. Experimental Verification

### 4.1. Control System Mode of Photoelectric Stabilized Platforms

**Figure 6:**
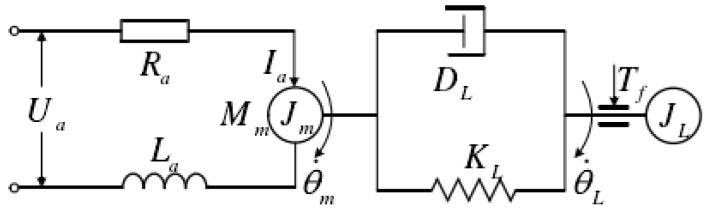
Scheme of the torque motor and loads.

The airborne optoelectronic platform adopts a direct drive method using a DC torque motor, which drives the frame to move through a rigid connection. The schematic diagram is shown in Figure 6.

Write the motion equations for the motor and load ends separately.

(33) $$J_{m}{\ddot{\theta }}_{m}(t)+D_{L}\left({\dot{\theta }}_{m}(t)-{\dot{\theta }}_{L}(t)\right)+K_{L}\left({\dot{\theta }}_{m}(t)-{\dot{\theta }}_{L}(t)\right)=T_{m}(t)$$

(34) $$J_{L}{\ddot{\theta }}_{L}(t)+D_{L}\left({\dot{\theta }}_{L}(t)-{\dot{\theta }}_{m}(t)\right)+K_{L}\left({\dot{\theta }}_{L}(t)-{\dot{\theta }}_{m}(t)\right)+T_{f}=0$$

Write equations for the motor:

(35) $$u_{a}=R_{a}i_{a}(t)+L_{a}i_{a}(t)+K_{e}{\dot{\theta }}_{m}$$

(36) $$T_{m}(t)=K_{t}i_{a}(t)$$

where $\theta_{m}$, ${\dot{\theta }}_{m}$, and ${\ddot{\theta }}_{m}$, respectively, refer to the rotation angle, angular velocity, and angular acceleration at the motor, and $\theta_{L}$, ${\dot{\theta }}_{L}$, ${\ddot{\theta }}_{L}$, respectively, refer to the rotation angle, angular velocity, and angular acceleration of the load. $T_{m}$ is the output torque of the motor, $J_{m}$ is the rotation inertia of the motor, $J_{L}$ is the rotation inertia of the load, $D_{L}$ is the system damping coefficient, $K_{L}$ is the system stiffness coefficient, $T_{f}$ is the frictional force, $u_{a}$ is the input voltage of the motor, $R_{a}$ is the armature resistance, $i_{a}$ is the armature current of the motor, $L_{a}$ is the armature inductance of the motor, and $K_{e}$ is electromotive force. For the convenience of analysis, it was assumed that the connection between the motor and the load was purely rigid and that $\theta_{m}=\theta_{L}$.

(37) $$\frac{{\dot{\theta }}_{L}}{u_{a}-\frac{R_{a}}{K_{t}}T_{f}}=\frac{K_{t}}{J_{\sum }\left(L_{a}s^{2}+R_{a}s\right)+K_{t}K_{e}}$$

Without considering the back electromotive force and armature inductance, and with relatively low friction force,

(38) $${\dot{\theta }}_{L}(s)=\frac{k_{m}}{s}\left(u(s)-bT_{f}(s)\right)=\frac{k_{m}}{s}\left(u(s)-d_{f}(s)\right)=\frac{k_{m}}{s}u(s)$$

where $k_{m}=\frac{K_{t}}{J_{\sum }R_{a}}$ is the controlled object, approximated as a first-order pure integral.

### 4.2. Application of the Proposed ESO + KF Control

When considering disturbance, the equation can be described as the following:

(39) $$\left\{\begin{array}{l} {\dot{x}}_{1}=k_{m}(u+d)\\ y=x_{1} \end{array}\right.$$

where the control variable u is the system input, d is the equivalent system lumped disturbance, and the lumped disturbance action is transformed into a new state variable. This results in an extended state equation of the following:

(40) $$\left\{\begin{array}{l} {\dot{x}}_{1}=k_{m}(u+x_{2})\\ {\dot{x}}_{2}=a(t)\\ y=x_{1} \end{array}\right.$$

where a(t) is the differentiation of the lumped disturbance. The observer for the extended state can then be designed as the following:

(41) $$\left\{\begin{array}{l} e_{1}=z_{1}-y\\ {\dot{z}}_{1}=z_{2}-\beta_{1}e_{1}+bu\\ {\dot{z}}_{2}=-\beta_{1}fal(e_{1},\frac{1}{2},\delta )\\ y=z_{1} \end{array}\right.$$

where $e_{1}$ is the error between the observed output and the actual output, $z_{1}$ is the observed value of the state variable, $z_{2}$ is the observation of the lumped disturbance, and $\beta_{1}$ and $\beta_{2}$ are the gains of the extended state observer.

A saturation function $fal(e_{1},b,\delta )$ can be used to suppress signal jitter, which can be described as the following:

(42) $$fal(e_{1},b,\delta )=\left\{\begin{array}{ll} e/\delta^{b-1} & \left|e\right|\leq \delta \\ {\left|e\right|}^{b}sign(e) & \left|e\right|>\delta \end{array}\right.$$

Considering $u=u_{0}-z_{2}$, the function is expressed as

(43) $$\left\{\begin{array}{l} {\dot{x}}_{1}=k_{m}(u+d-z_{2})\\ y=x_{1} \end{array}\right.$$

As long as the observer design is accurate, $z_{2}\to d$, $d-z_{2}\to 0$, and the influence of disturbance on the system can be eliminated.

The formula for calculating the ESO + KF based on the mathematical model of the airborne optoelectronic platform was derived from the above equation:

(44) $$\left\{\begin{array}{l} {\hat{x}}_{k}^{-}={\hat{x}}_{k-1}+Tk_{m}u_{k-1}\\ P_{k}^{-}=P_{k-1}+S+Q \end{array}\right.$$

(45) $$\left\{\begin{array}{l} K_{k}=\frac{P_{k}^{-}}{P_{k}^{-}+R}\\ {\hat{x}}_{k}={\hat{x}}_{k}^{-}+K_{k}(y_{k}-{\hat{x}}_{k}^{-})\\ P_{k}=P_{k}^{-}(1-K) \end{array}\right.$$

### 4.3. Methodology and Experimental Results

**Figure 7:**
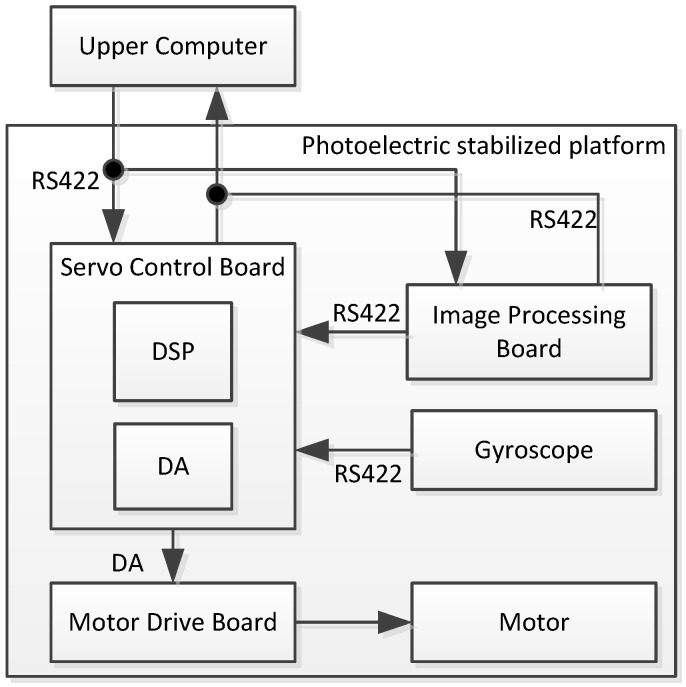
Scheme of the signal connection for the experiment.

The signal connection scheme of the experiment is shown in Figure 7. The experimental device is mainly divided into several components, including the upper computer, servo control board, motor drive board, image processing board, and gyroscope. The communication between each component uses an RS422 serial port. The upper computer sends control commands to the servo control board and image processing board. The image processing board target recognition unit collects image signals for target recognition from the image detector and outputs deviation signals after comparing them with the target position. The deviation signal indicates the distance that the line-of-sight has deviated from the target. When the deviation from the target is significant, it indicates that the line-of-sight has deviated from the target.

The servo control board calculates the servo control algorithm based on the upper computer instructions and the deviation target signal from the image processing card, combined with the feedback data of the gyroscope, and outputs the control amount to the drive board to drive the DC motor to move, keeping the deviation target signal at 0.

The gyroscope we used was an HPFG-3 three-axis MEMS gyroscope. The motor that was adopted was a brush DC motor model called NH225LYX-M30-E28.

**Figure 8:**
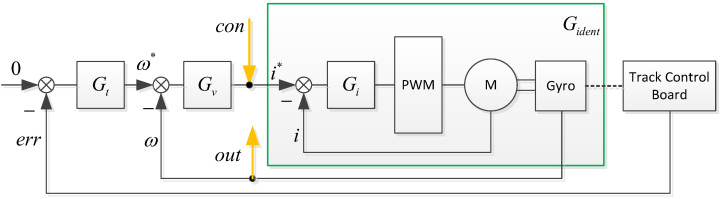
Control block diagram and the sweep frequency signals.

This article used the frequency sweep method to test and identify the system model. The servo system control block diagram is shown in Figure 8.

**Figure 9:**
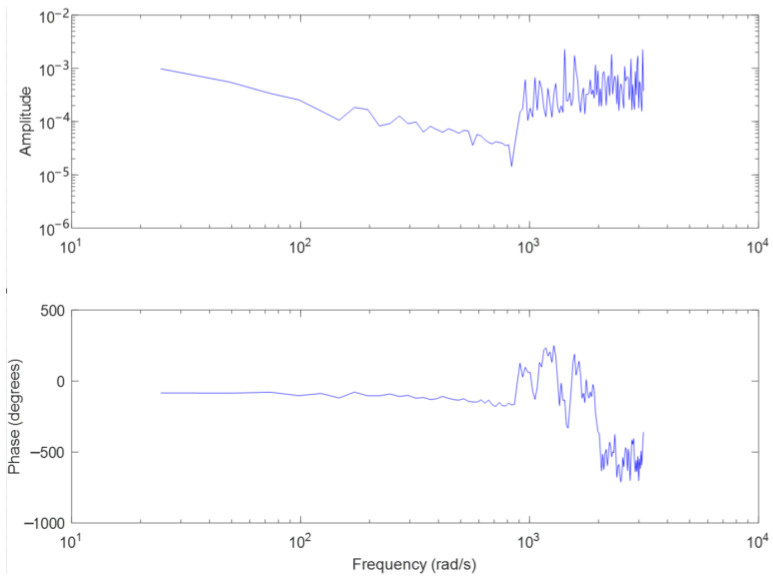
Bode diagram for identifying models.

$G_{t}$, $G_{v}$, and $G_{i}$ are the tracking loop, speed loop, and current loop controllers, respectively, $G_{ident}$ is the control model to be identified, err, ω and i are detected miss distance, speed, and current respectively, $\omega^{*}$ and $i^{*}$ are input values for speed and current loops. con is the input signal of the sweep frequency test, and out is the output signal of the sweep frequency test. con is a sine signal ranging from 0 Hz to 300 Hz, collecting the sweep frequency output signal, and using the MATLAB system identification toolbox for model identification, as shown in Figure 9. Without considering the mechanical resonance link, $k_{m}=0.023$.

**Figure 10:**
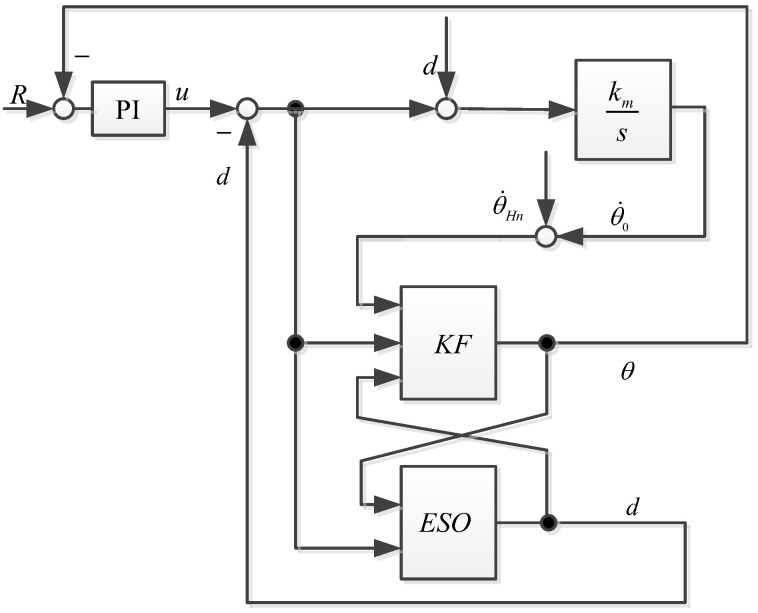
Proposed ESO + KF used in platform.

The ESO + KF algorithm proposed in this article, combined with the mathematical model of the stable platform, produced a control block diagram as shown in Figure 10.

**Figure 11:**
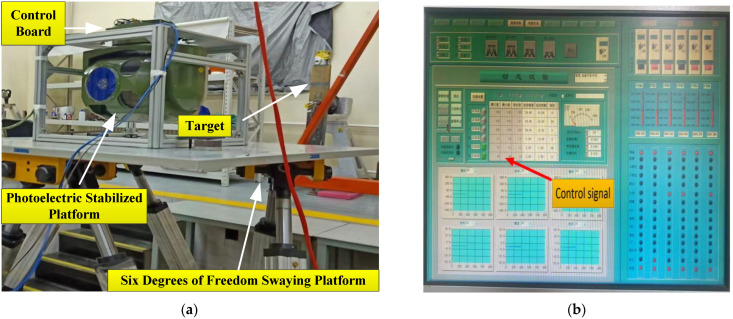
Laboratory experimental equipment and its driving signal settings: (**a**) the six degrees of freedom swaying platform and (**b**) the upper computer and control signals.

The experimental plan is shown in Figure 11. The airborne optoelectronic stabilization platform is connected to the upper computer through communication cables, and the upper computer sends instructions to control the stabilization platform. The target is a fixed circular light point, which is tracked by an optoelectronic stabilization platform. Fix When the stable platform is fixed on the hexapod swing platform, the hexapod swing platform reciprocates with a certain amplitude and period to simulate disturbances of different amplitudes and frequencies. The servo control board is used for algorithm calculation. The stronger the anti-interference ability of the servo algorithm, the smaller the deviation of the target from the center.

Firstly, static experiments were conducted to collect gyro angular velocity information to verify the effectiveness of ESO + KF in noise processing. The photoelectric platform and the six degrees of freedom swaying platform were both in a stationary state. Gyroscope angular velocity data was collected, and then a traditional 150 Hz low-pass filter and the ESO + KF algorithm were used to process the original gyroscope angular velocity data. The comparison of three sets of angular velocity data for unfiltered data, traditional low-pass filter filtering, and the ESO + KF algorithm is shown in the following figure. Due to the low bandwidth of traditional low-pass filters, it can cause serious lag and loss of gyroscope speed measurement information. When the bandwidth of traditional low-pass filters is too high, the noise-filtering effect is limited. Therefore, in this experiment, based on previous platform debugging experience, a traditional low-pass filter bandwidth of 150 Hz was selected.

**Figure 12:**
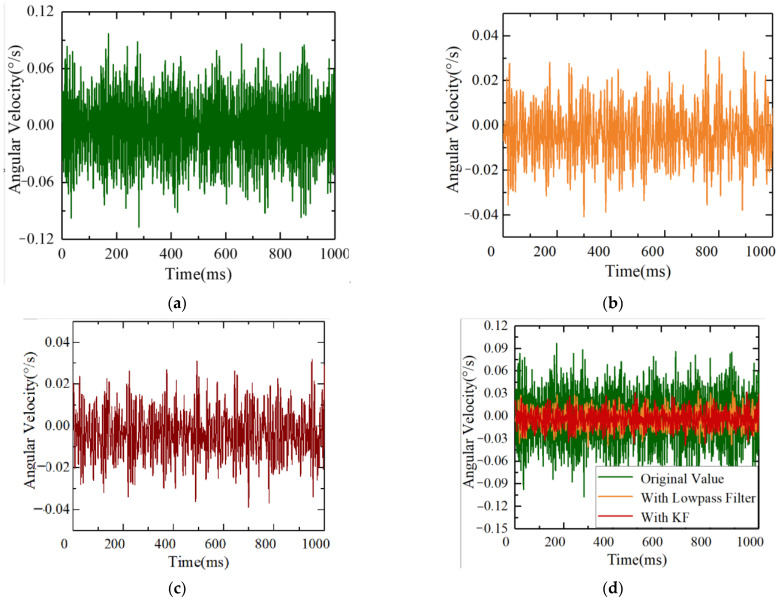
Acquisition of gyro angular velocity data under static conditions: (**a**) unprocessed gyroscope data; (**b**) gyro data with low-pass filtering; (**c**) gyro data with ESO + KF; and (**d**) a comparison of the three groups of data.

The original data collected by the gyroscope under a static state are shown in Figure 12. Due to the influence of electromagnetic interference and other factors, the static noise of the gyroscope was ±0.102°/s. The gyro noise after low-pass filtering is shown in Figure 12b, and the static noise was reduced to ±0.042°/s. Figure 12c shows the gyroscope data after using the Kalman filter. It can be seen from the figure that the noise was reduced to ±0.038°/s after Kalman filter application.

**Figure 13:**
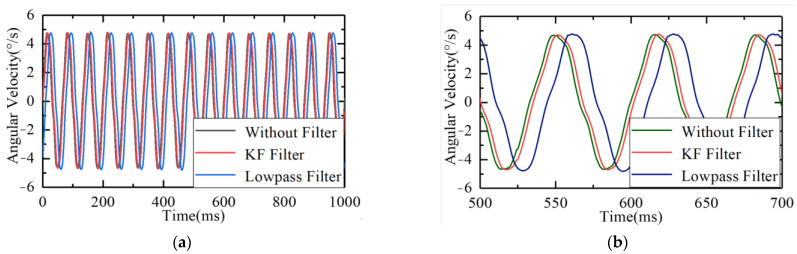
Comparisons of raw gyro, low–pass filter, and Kalman filter data: (**a**) general comparison and (**b**) local alignment.

The six degrees of freedom swaying platform was in a stationary state, and the control platform framework moved in a closed loop at 5°/15 Hz to collect the raw data of the gyroscope. Then, the raw information of the gyroscope was processed using 150 Hz low–pass filtering and the Kalman filter, respectively. The comparison of the three sets of data is shown in Figure 13.

As shown in the figure, whether using a Kalman filter or a 150 Hz low-pass filter, there will be a certain degree of lag in the original signal while filtering. Compared to traditional low-pass filtering, the Kalman filter had a relatively small lag and better real-time performance. From the point of view of the data, the lag of the Kalman filter was 0.12 cycles, and the lag of the low-pass filter was 0.27 cycles.

In summary, in terms of noise reduction, the Kalman filter has better noise-filtering effects and causes less lag. For airborne optoelectronic platforms, the real-time performance of the servo control system is very important. Serious lag will lead to reduced bandwidth, decreased disturbance suppression capability, and even cause instability in the system. Therefore, the Kalman filter is suitable for the servo control of airborne optoelectronic platforms, and it has better noise-filtering effects and real-time performance without increasing the hardware’s burden too much.

**Figure 14:**
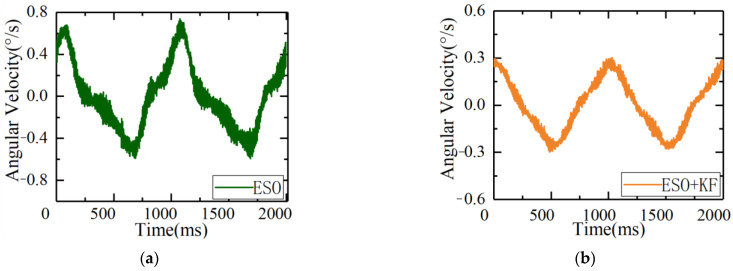

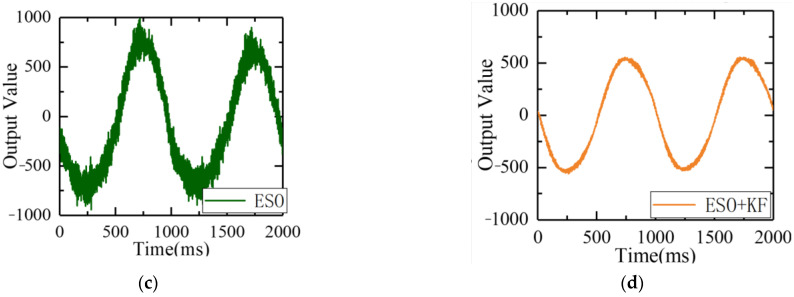
Comparison of speed fluctuation and output between the ESO and ESO + KF under the same disturbance source: (**a**) speed fluctuation of the ESO; (**b**) speed fluctuation of the ESO + KF; (**c**) output of the ESO under disturbance; and (**d**) output of the ESO + KF under disturbance.

To verify the effectiveness of the ESO + KF disturbance suppression, a dynamic experiment was conducted by starting the hexapod swing platform and simulating the disturbance imposed on the inertial platform by the external environment with a motion of 1°1 Hz. The inertial platform maintained spatial stability, with the velocity loop input set to 0. Under this condition, the comparison of the disturbance suppression capabilities of the ESO + KF and traditional ESO proposed in this article is shown in Figure 14.

As shown in Figure 14, under the same disturbance conditions, the ripple of error of the servo control system using the traditional ESO algorithm was ±0.72°/s, while the ripple of error using the ESO + KF algorithm was 0.28°/s. It can be seen that the ESO + KF improved the disturbance suppression capability of the servo control system. By comparing the controller outputs of the two methods, it can be concluded that due to the influence of gyro noise, the driving amount using the ESO control method rippled greatly, causing severe burden on the controller and even resulting in system resonance in severe cases. When using the ESO + KF control algorithm, the fluctuation of the controller was reduced under the same bandwidth of the ESO controller with the same gain, which reduced the burden on the controller and further improved the gain of the ESO under this condition.

**Figure 15:**
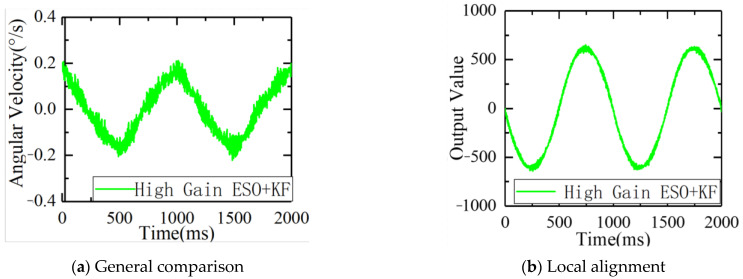
Speed fluctuation and output of the high-gain ESO + KF under disturbance: (**a**) comparison of the speed fluctuation and (**b**) comparison of the output.

Under the same disturbance conditions on the six degrees of freedom swaying platform, the remaining parameters of the servo control loop remained unchanged, and the gain of the ESO controller was increased. The speed fluctuations and driver outputs are shown in Figure 15.

As can be seen from the figure, after further increasing the gain, the ripple of errors of the ESO + KF was further reduced to 0.19°/s, further improving its disturbance suppression capability.

**Figure 16:**
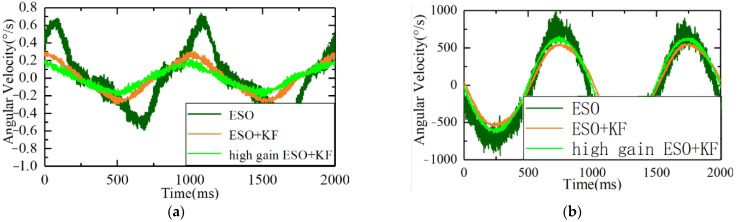
Comparison of speed fluctuation and output between the ESO, ESO + KF, and high-gain ESO + KF under the same disturbance source: (**a**) comparison of the speed fluctuation and (**b**) comparison of the output.

From observing Figure 16 and Table 1, it can be concluded that under the condition of a 1°1 Hz swing, with the same gain, the ESO + KF stationary speed errors were reduced from 0.72°/s to 0.28°/s, and the standard deviation was reduced from 0.32°/s to 0.17°/s. The results show that the ESO + KF had better immunity performance, and the high-gain ESO + KF can further reduce stationary errors to 0.19°/s and the standard deviation to 0.11°/s. The high-gain ESO + KF algorithm has a faster response speed while reducing the burden on the controller. It can be concluded that the ESO + KF can reduce the noise of the gyroscope, while also improving the immunity performance and reducing the burden on the controller, which can further improve the ESO’s gain and immunity level.

To further verify the improvement of the ESO + KF on the level of LOS stabilization, a dynamic experiment was conducted. The electro-optical stabilization platform was fixed on the six degrees of freedom swaying platform, and a parallel light tube simulated a point target at infinity in space. The stabilization platform was made to track the star target emitted by the light tube. The off-target quantity under image tracking represented the number of image pixels that deviated from the target along the LOS. The six degrees of freedom swaying platform was controlled to move at 1°/1 Hz and 3°/1 Hz, respectively, to apply disturbances to the platform. The real-time off-target quantity was used to observe the LOS stabilization accuracy under different control algorithms.

**Figure 17:**
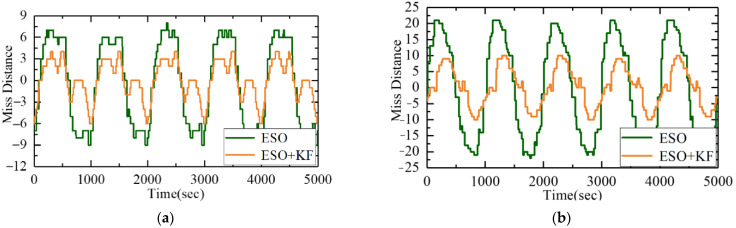
Comparison of off-target quantities between the ESO and ESO + KF under different disturbances: (**a**) disturbance of 1°/1 Hz and (**b**) disturbance of 3°/1 Hz.

Figure 17 shows the ripple of error and off-target quantity curves of the traditional ESO and ESO + KF control systems under 1°/1 Hz swing conditions. Figure 17 shows the target offset and off-target quantity curves of the traditional ESO and ESO + KF control systems under 3°/1 Hz z swing conditions. Table 2 and Table 3 show the statistical values of error fluctuations under 1°1Hz and 3°1Hz conditions, respectively. From the Table 2 and Table 3, it can be concluded that at 1°/1 Hz, the standard deviation of the traditional ESO off-target quantity was 6.072, and the standard deviation of the off-target quantity using the ESO + KF control system was 2.721. The stability accuracy level increased by 44.81% under the same rolling conditions. At 3°/1 Hz, the standard deviation of the traditional ESO was 15.60, and the standard deviation of the off-target quantity using the ESO + KF control system was 6.15, which improved the stability accuracy level by 39.42%. The experiments have shown that under different swing conditions, the ESO + KF can achieve better control system stability accuracy levels.

## 5. Conclusions

After establishing that the problem that the gyro noise of airborne electrooptical stabilization platform is serious, reduces the accuracy of ESO observation disturbance, and leads to a poor LOS stabilization effect, this paper proposed a disturbance suppression algorithm combining a Kalman filter and ESO to reduce the influence of transmission gyro noise and external interference torque on LOS stabilization accuracy and realize long-distance and long-focus tracking. Firstly, the disturbance observed by the ESO was compensated by a Kalman filter mathematical model, and the state variables observed by the Kalman filter were updated to the disturbance observed by the ESO for further observation. Then, an iterative control model combining the ESO and KF was derived, and the control algorithm was applied to the airborne optoelectronic platform to experimentally verify the accuracy of the line-of-sight stability under different forms of disturbance signals. The results showed that the stability accuracy increased by 44.81% under 1°/1 Hz swing conditions and 39.42% under 3 °/1 Hz swing conditions. Finally, through the installation of a flight verification, the photoelectric platform was loaded on a helicopter, and the new control algorithm could reduce external environmental disturbances and maintain the stability of the line-of-sight, further proving the effectiveness of the algorithm and providing theoretical support for high-precision disturbance suppression in high-noise environments.

## Figures and Tables

**Table 1:** Comparison of speed fluctuation between the ESO, ESO + KF, and high-gain ESO + KF.

Method	Range	Std.
ESO	±0.72°/s	0.32°/s
ESO + KF	±0.28°/s	0.17°/s
High Gain ESO + KF	±0.19°/s	0.11°/s

**Table 2:** Comparison of off-target quantity between the ESO and ESO + KF under 1°1 Hz.

Method	Ripple of Errors	Standard Deviation
ESO	±9	6.02
High-Gain ESO + KF	±6	2.71

**Table 3:** Comparison of off-target quantity between the ESO and ESO + KF under 3°1 Hz.

Method	Ripple of Errors	Standard Deviation
ESO	±21	15.60
High-Gain ESO + KF	±9	6.15

## Data Availability

Data are contained within the article.
